# Recurrent ilioinguinal lymph node metastasis from primary anal adenocarcinoma: what should we do? – A case report and review of literature

**DOI:** 10.1016/j.ijscr.2020.05.020

**Published:** 2020-05-21

**Authors:** Y. Zhao, R. Wijaya

**Affiliations:** Department of General Surgery, Changi General Hospital, 2 Simei Street 3, S529889, Republic of Singapore

## Abstract

**Introduction:**

Anal canal tumors are uncommon amongst gastrointestinal tumors or anorectal tumors. While the majority of them seem to be squamous cell carcinoma in nature, adenocarcinoma may be equally as common amongst the Asian population. Recurrent nodal metastasis from a primary anal malignancy is not a rare occurrence in view of the anatomy of the anal canal.

**Case presentation:**

A 70 year-old patient underwent surgery for synchronous sigmoid and anal adenocarcinoma in 2015. He then re-presented 2 years later with recurrence in the right inguinal lymph nodes. He subsequently underwent a right ilio-inguinal lymph node block dissection with a Sartorius flap creation.

**Discussion:**

As most anal canal tumors are squamous cell carcinomas, the optimal treatment for recurrent ilioinguinal lymph node disease has been well-established. This usually involves groin dissection as surgical treatment, with consideration for adjuvant combined chemoradiotherapy. Such an approach is likely to be beneficial for ilioinguinal lymph node disease from primary anal canal adenocarcinomas as well.

**Conclusion:**

Physicians caring for patients with primary anal adenocarcinoma should be vigilant for possible ilioinguinal lymph node metastasis as this is not a rare occurrence. Surgical treatment appears to be a reasonable approach, with consideration for adjuvant therapy.

## Highlights

• Anal adenocarcinoma is more common in the Asian population than previously thought.
• Treatment of ilioinguinal lymph node metastasis from anal canal squamous cell carcinoma has been well described, but the management for primary adenocarcinoma is less so.
• Groin dissection for recurrent ilioinguinal lymph node metastasis from primary anal adenocarcinoma is a reasonable approach.

## 1 Introduction

Anal canal tumours are uncommon, representing less than 1% of tumours along the gastrointestinal tract [1], and around 5% of all anorectal cancers [2]. While literature from the West reports a majority being squamous cell carcinoma, recent Asian literature seems to show that adenocarcinoma as a pathological diagnosis could be equally as common, if not more common [3], [4].

In view of the anal canal anatomy, recurrent nodal metastasis from a primary anal malignancy to the inguinal lymph nodes is not a rare occurrence, with literature reporting prevalence of 10%–17% for recurrent nodal disease [5], [6], [7]. It has also been reported that the presence of inguinal lymph node disease in anal adenocarcinomas at diagnosis could be a poor prognostic factor, with lower 5-year survival rates [7]. Unfortunately, the low incidence of anal adenocarcinoma as described earlier results in limited data and studies and thus, a paucity in evidence-based recommended treatment modalities as compared to squamous cell carcinomas. Given that the subset of patients with anal adenocarcinoma and recurrent inguinal lymph node disease is even smaller; such information is not widely reported.

We hereby present a case of a patient with synchronous sigmoid and anal adenocarcinoma that had previously undergone a laparoscopic abdominoperineal resection with left para-aortic lymph node, but then recurred with inguinal lymph node disease 2 years later [8]. We will also perform a review of the literature and current evidence of suitable management options for ilio-inguinal lymph node disease for anal adenocarcinomas. This work has been reported in line with the SCARE criteria [9].

## 2 Presentation of case

A 70 year-old Chinese gentleman presented to our centre in 2015 with an anal mass, on the background of a chronic fistula-in-ano, that had been growing in dimensions for the past 5 years, associated with per-rectal bleeding, tenesmus and reduced stool calibre of the past 2–3 years as well as loss of weight of 10 to 15 kg. He was worked up for this and was diagnosed to have synchronous anal and sigmoid adenocarcinoma. He underwent a laparoscopic abdominoperineal resection and left para-aortic lymph node clearance, gluteal mobilization and primary closure in 2015 for the disease. [7] The final histological diagnosis came back as T3N0M0 anal adenocarcinoma and T3N1M0 sigmoid adenocarcinoma. He was recommended for adjuvant chemoradiotherapy and declined despite extensive counselling.

Post operatively, he has been followed-up every three months in our specialist outpatient clinic with thorough history taking, physical examination and measurements of his carcinoembryonic antigen (CEA) levels. He also underwent surveillance computed tomography scans of the chest, abdomen and pelvis (CT-TAP) regularly. The surveillance CT-TAP on September 2017 reported an interval increase in size of the right inguinal lymph node with new calcific foci, suspicious for metastatic adenopathy. No other evidence of local recurrence or distant metastasis was noted. Clinically, a 2 cm right inguinal lymph node could be palpated at the medial aspect of the right groin. In view of this finding, the patient underwent an ultra-sound guided fine needle aspiration of the right inguinal lymph node for cytological analysis. The cytology came back as mucinous adenocarcinoma consistent with metastases from known anal primary. A colonoscopy through his end-colostomy was arranged for the patient as well, with no significant findings noted. CEA was not elevated. The disease-free interval was 27 months.

Chemoradiotherapy was once again brought up as a possible treatment modality, but the patient was adamantly against it despite extensive counselling. In view of isolated loco-regional recurrence to the right inguinal lymph node, consideration of right ilio-inguinal lymph node dissection was made. The patient was counselled on the procedure, as well as the possible complications and he agreed to the procedure.

**Fig. 1:**
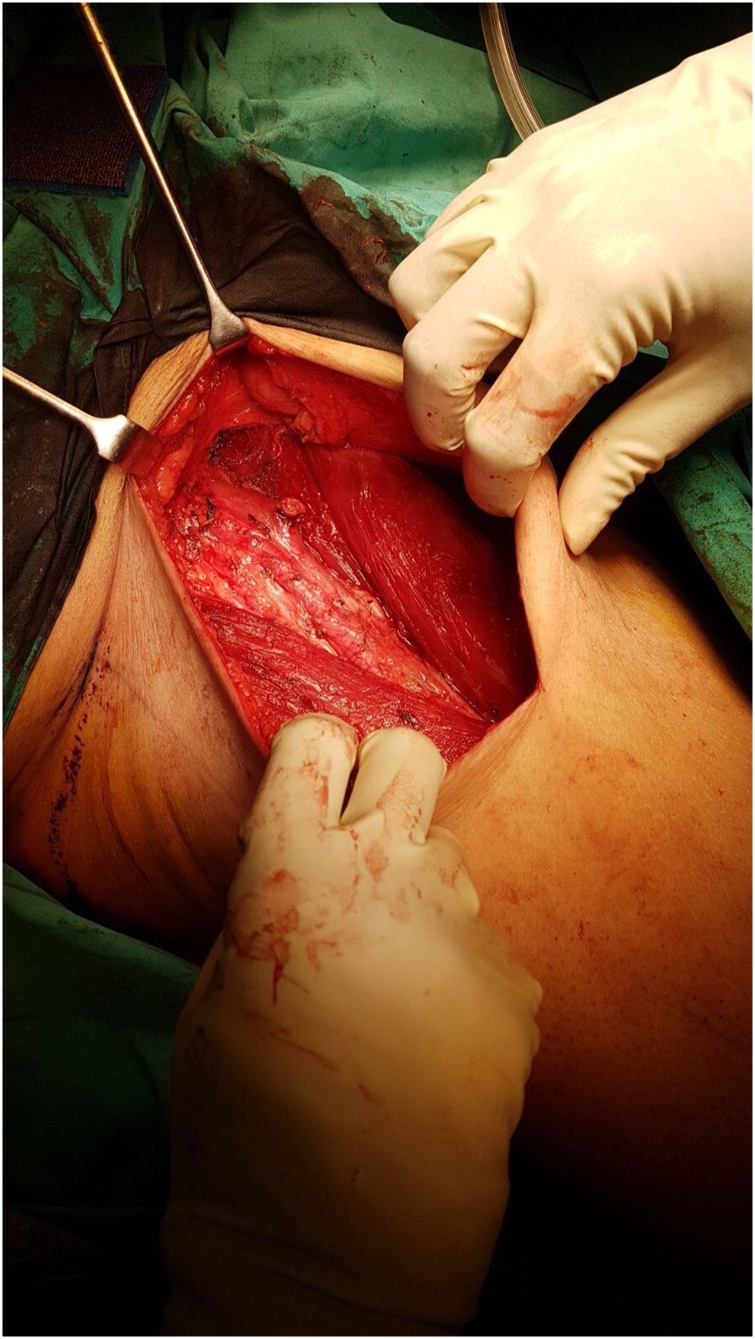
Identification of femoral canal.

The patient underwent a right ilioinguinal lymph node block dissection with Sartorius flap creation in November 2017. He was positioned supine with his right leg abducted and externally rotated. The femoral triangle boundaries were identified and an ellipitical skin incision was made over the mid-point. The great saphenous vein was then ligated distally at the apex of the femoral triangle, while the right femoral canal contents were identified and preserved (Fig. 1). The inguinal lymph nodes and Cloquet's nodes was taken en-bloc. The femoral canal was then repaired before mobilisation of the Sartoius flap and tagging of the flap over the femoral vessels. 2 drains were placed on the wound prior to skin closure.

Histopathological analysis of the surgical specimen identified the presence of 18 lymph nodes, of which 1 was positive for metastatic mucinous adenocarcinoma.

The patient recovered well post-operatively. His case was discussed at a post-operative multi-disciplinary tumour board meeting, which recommended adjuvant combined modality therapy for which he has once again declined. He is currently 2 years disease free survival.

## 3 Discussion

The optimal treatment for recurrent ilioinguinal lymph node metastasis from anal squamous cell carcinomas has been well-established. The National Comprehensive Cancer Network (NCCN) has established that for patients with complete remission after initial therapy, inguinal node palpation and imaging of the pelvic region should be done on a regular basis for follow-up. Should there be a recurrence in the inguinal lymph nodes, groin dissection would be the recommended surgical option. There should also be consideration for adjuvant combined modality therapy, especially if there has been no previous radiotherapy to the groin region. The benefits of this approach can be seen in the retrospective study by Gerard et al. [10]. It reported a study of 270 patients with anal canal carcinomas, 46 of whom had inguinal lymph node disease. They then underwent inguinal lymph node dissection and adjuvant radiotherapy, and the 5-year survival was reported at 54.4% and 41.4% for patients with concurrent and recurrent lymph node disease respectively.

While the most common histopathological pattern for anal malignancies worldwide is squamous cell carcinoma, it has been reported in Asian studies that adenocarcinoma could be the more common subtype instead, with Sun et al. [4] quoting a prevalence of 71.4% over 21 years and 4 institutions. A recent study by Su et al. [6] shed more light into primary anal adenocarcinomas, with 5-year inguinal recurrence quoted at 17%. An earlier study by Li et al. [7] reported a figure of 16.7%. It also showed that patients with positive inguinal lymph node disease are likely to have a poorer survival outcome than patients without (19.1% vs 46.9%). The publication of such literature from Asia does seem to raise questions not only about the potential beneficial role of prophylactic ilio-inguinal lymph node dissection but also the role of early detection of ilio-inguinal lymph node disease.

Sentinel lymph node biopsies have been studied as a possible way for earlier detection of ilioinguinal lymph node disease in anal squamous cell carcinomas. A systematic review on this was performed by Noorani et al in 2013, identifying 17 studies with 270 patients. The estimated false negative rate was quoted as between 0 to 18.75% [11]. It concluded that SLN biopsy can be a feasible method for detection of inguinal node metastasis, to identify patients who will benefit most from groin radiotherapy. This stems from the need for accurate diagnosis of ilioinguinal lymph node disease in view of the multiple side effects of prophylactic groin radiotherapy, which was previously used.

The management for recurrence of ilioinguinal lymph node disease from anal adenocarcinomas specifically however is less clear cut and lacks guidance from society guidelines. Thus, it would seem that performing a groin dissection for recurrent lymph node disease could be a reasonable approach for anal adenocarcinoma, similar to how anal squamous cell carcinomas should be managed optimally. Primary physicians should also consider adjuvant chemoradiotherapy, especially if there has been no previous radiotherapy to the groin area.

## 4 Conclusion

In conclusion, ilioinguinal lymph node metastasis from a primary anal adenocarcinoma can be expected given the anatomy of the lymphatic drainage from the anal canal. Surgical treatment should be the treatment of choice given the survival outcomes as reported by prior studies, with consideration for adjuvant chemoradiotherapy as well.

## Competing interest

No benefits in any form have been received or will be received from a commercial party related directly or indirectly to the subject of this article.

## Funding

None.

## Ethical approval

Because this is a case report, the present study was not appreciated by a research ethics committee. However, written informed consent was obtained from the patient for publication of this case report.

## Consent

Written informed consent was obtained from the patient for publication of this case report and accompanying images. A copy of the written consent is available for review by the Editor-in-Chief of this journal on request.

## Author contribution

Zhao Y: investigation, writing - original draft, writing - review & editing Ramesh W: conceptualization, methodology, writing - review & editing, resources, supervision.

## Registration of research studies

The present study is not a research involving humans, but a clinical case report, whose patient authorized the publication by means of a free and informed consent term.

## Guarantor

Zhao Y, Ramesh W.

## Provenance and peer review

Not commissioned, externally peer-reviewed.
